# To Be or Want to Be: Disentangling the Role of Actual versus Ideal Self in Implicit Self-Esteem

**DOI:** 10.1371/journal.pone.0108837

**Published:** 2014-09-30

**Authors:** Jonathan Remue, Sean Hughes, Jan De Houwer, Rudi De Raedt

**Affiliations:** 1 Department of Experimental Clinical and Health Psychology, Ghent University, Ghent, Belgium; 2 Department of Psychology, National University of Ireland Maynooth, Maynooth, Ireland; Swansea University, United Kingdom

## Abstract

A growing body of work suggests that both depressed and non-depressed individuals display implicit positivity towards the self. In the current study, we examined whether this positivity can be underpinned by two qualitatively distinct propositions related to actual (‘*I am good*’) or ideal (‘*I want to be good*’) self-esteem. Dysphoric and non-dysphoric participants completed a self-esteem Implicit Association Test (IAT) as well an Implicit Relational Assessment Procedure (IRAP) targeting their actual self-esteem and an IRAP targeting ideal self-esteem. Both groups demonstrated similar and positive IAT effects. A more complex picture emerged with regard to the IRAP effects. Whereas non-dysphorics did not differ in their actual and ideal self-esteem, their dysphoric counterparts demonstrated lower actual than ideal self-esteem. Our results suggest that closer attention to the role of propositional processes in implicit measures may unlock novel insight into the relationship between implicit self-esteem and depression.

## Introduction

Self-esteem has been extensively investigated by researchers from a wide variety of theoretical persuasions and currently represents a key explanatory construct in many areas of psychological science, including health psychology [1], social psychology [2], [3] and clinical psychology [4]. Within the latter domain, negative self-schemas are thought to bias information processing in an automatic, repetitive and difficult to control manner [5]. These negative cognitions about the self are also argued to play a significant role in the maintenance and recurrence of depressive episodes [6], [7]. Interestingly, however, much work on self-esteem and its relationship to depression has employed self-report measures which are susceptible to a variety of response biases such as social desirability and self-presentation. Many cognitive models of depression also assume that self-related schemata are not always consciously accessible and thus cannot always be verbally reported upon [8], [9]. Consequently, it is questionable whether the use of self-report measures may provide meaningful information about such schemata. To overcome these limitations, a number of alternative procedures have recently emerged that reduce the participant's ability to control their responses and operate in such a way that they do not depend on introspective access to the psychological content of interest. Whereas self-report measures of self-esteem can be classified as explicit measures that capture non-automatic instances of self-evaluation (e.g., self-evaluations that occur when participants have ample time and resources to reflect or have the intention to evaluate the self), implicit self-esteem measures can be thought of as measures that register more spontaneous, automatic self-evaluations (e.g., self-evaluations that occur quickly or when participants do not have the intention to evaluate the self; see [10]).

Interestingly, a growing literature suggests that although depressed and non-depressed people differ with respect to their explicit self-esteem they demonstrate surprisingly similar levels of (positive) implicit self-esteem [11], [12], [13], [14]. Consider, for example, the work of De Raedt and colleagues [11] who compared implicit self-esteem in a group of depressed participants relative to healthy controls using three separate paradigms: the Implicit Association Test (IAT), Name Letter Preference Task (NLPT), and the Extrinsic Affective Simon Task (EAST). Across all three measures evidence for similar levels of positive implicit self-esteem was obtained for both groups. Some studies have even reported higher levels of (positive) implicit self-esteem in formerly depressed relative to never-depressed participants [15], [16].

In an attempt to explain these surprising findings, De Raedt and colleagues [11] argued that the IAT and other measures of implicit self-esteem may have captured *actual* self-esteem in non-depressed participants but *ideal* self-esteem in depressed participants. Whereas actual self-esteem refers to feelings of self-worth or the global evaluation of the current self [17], ideal self-esteem is considered to be a global representation of the attributes a person would like to possess (see [18]). Numerous studies have provided compelling evidence for the role of discrepancies between ideal and actual self in depressive disorders [19], [20]. One way to conceptualize actual and ideal self-esteem is in terms of the type of relation between the self and positive and negative valence. One could argue that both actual and ideal self-esteem involve such a relation but differ in the way that these concepts are related. Whereas actual self-esteem refers to current beliefs about the self (i.e., *I am* good/bad), ideal self-esteem would reflect beliefs about the desired future self (i.e., I *want to be* good/bad). These beliefs are propositional in nature because, unlike associations, they contain information about how concepts are related (see [21], for an excellent discussion of the core differences between propositions and associations).

De Raedt and colleagues' [11] hypothesis certainly seems plausible given implicit measures are usually designed to assess whether one set of concepts (e.g., ‘self’ and ‘other’) is somehow related to a second set of concepts (e.g., ‘positive’ or ‘negative’) without regard to the way in which those concepts are related. To illustrate, consider a typical self-esteem IAT. During a first test phase, participants categorize items related to the self (e.g., the first name of the participant) and positive words (e.g., HAPPY) using one response key and items related to someone else (e.g., the first name of another participant) and negative words (e.g., INCOMPETENT) using another response key. During a second test phase, response mappings are reversed so that self-related items and negative words are assigned to the first key whereas other-related items and positive words are assigned to the second key. The difference in how well someone performs during the first relative to the second phase is considered to provide an overall measure of how readily this person associates the concept “self” with positive or negative valence. However, an IAT effect does not reveal how a person relates those concepts. For some individuals, the IAT score might reflect the extent to which someone believes that he or she *is* good (i.e., actual self-esteem) whereas for other individuals, the same score might reflect that he or she *wants to be* good (i.e., ideal self-esteem).

With this idea in mind, Remue and colleagues [22] set out to distinguish actual and ideal implicit self-esteem using a relatively new procedure known as the Implicit Relational Assessment Procedure (IRAP; [23]). The IRAP stems from an intellectual tradition known as Contextual Behavioral Science [24] and a functional account of human language and cognition known as Relational Frame Theory (RFT; [25]). Unlike many other implicit measures, the IRAP was specifically designed to capture how objects, stimuli and events are automatically related to one another (i.e., what RFT researchers refer to as ‘brief and immediate relational responses’; see [26], [27]). If we assume that the ease with which people automatically relate stimuli is mediated by propositional knowledge in memory (see [28] for an in-depth discussion), it could be argued that performance on the IRAP provides an implicit measure of propositional knowledge. In order to test this assumption, Remue et al. exposed a group of dysphoric and non-dysphoric participants to two separate IRAPs: one designed to assess actual and another to assess ideal self-esteem. Consistent with their predictions, two contrasting patterns of implicit self-esteem emerged, with dysphoric participants showing evidence of lower actual and higher ideal self-esteem relative to their non-dysphoric counterparts who showed evidence of higher actual and lower ideal self-esteem compared to the former group. These results tentatively suggest that the implicit measures used by De Raedt and colleagues [11] may have assessed ideal self-esteem in the dysphoric group and actual self-esteem in the non-dysphoric group.

The present study set out to extend the work of De Raedt and colleagues [11] and Remue and colleagues [22] in several ways. Within the context of self-esteem, we examined whether implicit measures that are designed to capture associations may in fact reflect the operation of qualitatively distinct sets of propositions. Whereas De Raedt and colleagues only used an IAT and Remue et al. only used IRAPs, we asked our participants to complete both a self-esteem IAT and two separate IRAPs, one targeting actual (‘*I am*’) and another targeting ideal self-evaluations (‘*I want to be*’). Moreover, we pre-selected participants who reported either high scores (i.e., dysphoric group) or low scores (i.e., non-dysphoric group) on an index of depressive symptoms during an earlier screening study. Based on the ideas of De Raedt and colleagues [11], we expected contrasting patterns of implicit self-esteem as a function of the task employed and group tested. Although we expected dysphoric and non-dysphoric participants to produce similar (positive) scores on the self-esteem IAT, we anticipated that they would diverge in their respective IRAP performances, with the former group showing stronger ideal relative to the actual implicit self-esteem and the latter group showing stronger actual relative to ideal self-esteem. Furthermore, based on the idea the IAT might capture different aspects of self-esteem in dysphoric than in non-dysphoric participants, we expected that the IAT would correlate most strongly with the ideal self-esteem IRAP in the dysphoric group but with the actual self-esteem IRAP in the non-dysphoric group. In addition, we included a number of questionnaires to investigate whether a discrepancy between actual and ideal self-esteem would also emerge at the explicit level. Our goal here was to explore how implicit and explicit self-esteem interact within and between these two groups.

Finally, it is worth noting that the current study provided us with an opportunity to address three methodological issues that arose in our earlier work. First, Remue and colleagues [22] employed a shortened version of the IRAP containing two (rather than the standard of six) test blocks which may have adversely affected the reliability of the observed effects (see [29]). In order to circumvent this concern, and facilitate a direct comparison between our results and those observed elsewhere in the literature, the current study included a standard (six-block) version of the IRAP. Second, while Remue and colleagues [22] required participants to respond with both speed (2500 ms) and accuracy (80%) during the IRAP, recent evidence suggests that introducing even stricter mastery criteria could lead to more robust IRAP scores [26]. Hence, we opted for a more stringent set of latency criteria than before. Third and finally, although many of the stimuli used in Remue and colleagues were related to self-esteem several were more directly relevant to depression in general (e.g., “Happy”, “Sad”). Unlike the IAT in which the definition of the categories (e.g. ‘*Me*” and ‘*Worth*’) appears to be more important than the individual stimuli used (e.g. ‘*Peter*’ and ‘*Successful*’) [30], it is crucial that stimuli directly relevant to the domain of interest be employed in the IRAP (see [31] for a discussion). Therefore in the current study we only included items that were directly related to self-esteem.

## Materials and Methods

### Ethics statement

Participants gave their written informed consent and received either credit or €10 for their participation. The study was approved by the ethics committee of Ghent University. The investigation was conducted in full accordance with the principles expressed in the Declaration of Helsinki.

### Participants

Sixty-four students participated in the current study. Prior to the study, they were screened for depressive symptomatology using the BDI-II-NL [32]. These same participants completed the BDI-II-NL for a second time upon arriving at the laboratory for the actual test session. Both BDI-II-NL (pretest and test) scores correlated highly, and were based on the same high/low classifications. Using the recommended cut-off score from the BDI-II-NL manual, the final sample was divided into two groups: a low BDI group (≤13) consisting of 35 students (30 women and 5 men) ranging from 18 to 30 years (*M* = 21, *SD* = 2.84) and a high BDI group (≥14) consisting of 29 students (25 women and 4 men) ranging from 18 and 25 years (*M* = 19.38, *SD* = 2.06). Assignment to BDI groups was based on the BDI score during the second (test) session. By design, the high BDI group had significantly higher scores during test (*M* = 21.93, *SD* = 8.36) compared to the low group (*M* = 4.8, *SD* = 3.72), *t*(62) = 10.91, *p*<.001. Note that, by design, BDI scores during the test session were not normally distributed (Shapiro-Wilk = .892; *p*<.001) due to the fact that we invited participants with extremely high or low BDI scores during initial screening. We therefore used BDI as a dichotomous rather than continuous variable in our analyses (however, for a critical discussion see [33]).

### Measures

#### Beck depression inventory (BDI-II-NL)

The BDI-II-NL, a 21 item self-report inventory, was used to measure the severity of depressive symptoms [34]. The Dutch translation of the BDI-II has shown high internal consistency: Cronbach's α of.92 for a patient population and.88 for a healthy control group. Also, the validity index satisfies general psychometric criteria [27].

#### Rosenberg self-esteem scale

(RSES, [35]; Dutch translation by [36]). This self-report scale measures global feelings of self-worth or self-acceptance and is widely used because of its proven validity and test-retest reliability. It consists of 10 items where participants have to state whether they totally agree, agree, disagree or totally disagree with the presented statement. The overall score represents the degree of global self-esteem, with higher scores indicating higher self-esteem.

#### Semantic differentials

Participants were presented with the same twelve target stimuli as used in the IRAP and IAT (six positive and six negative) and asked to evaluate each of them using a five-point scale ranging from 0 (Totally Disagree) to 4 (Totally Agree). Each word was rated twice, once with respect to actual self-evaluations (e.g., ‘*I am successful*’) and once with respect to ideal self-evaluations (‘*I want to be successful*’). In this way we sought to acquire two broad measures of self-esteem, one related to self-reported actual (SR Actual) and a second related to self-reported ideal (SR Ideal) self-esteem. Finally, participants were given a number of additional questionnaires related to their psychological flexibility and rumination. However, all of these served exploratory purposes and will not be discussed further.

#### IAT

During the IAT, the words ‘*Me*’ and ‘*Not Me*’ served as the target category labels and the words ‘*Worth*’ and ‘*Worthless*’ served as the attribute category labels. Six positively valenced (the Dutch words for confident, nice, successful, important, intelligent, competent and pleasant) and six negatively valenced Dutch adjectives (insecure, inferior, failure, worthless, useless and stupid) served as attribute stimuli. The participant's first name and surname, place of residence and nationality were used as stimuli for the target category *‘Me’*. The first name and surname of another participant were used as two items for the target category *‘Not me’* while a fabricated (non-Belgian) place of residence and nationality were used as two additional items in that same category.

Prior to the onset of the IAT, participants were informed that a series of words would appear one-by-one in the middle of the screen and that their task was to categorize those stimuli as quickly and accurately as possible. They were also informed that the category labels ‘Me’ and ‘Not Me’ as well as ‘Worth’ and ‘Worthless’ would appear on the upper left and right sides of the screen and that stimuli presented in the middle of the screen should be assigned to these categories by pressing either the E (left response) or the I key (right response) on an AZERTY keyboard. Each trial started with the presentation of a fixation cross for 200 ms in the middle of the screen followed immediately by a target or attribute stimulus. If the participant categorized a word correctly - by selecting the appropriate key for that block of trials - the stimulus disappeared from the screen and the next trial began. In contrast, an incorrect response resulted in the presentation of a red ‘X’ which remained on-screen until the correct key was pressed. Overall, each participant completed seven blocks of trials. During the first block of 20 practice trials they were requires to sort the self- or other-related words into their respective categories, with ‘Me’ assigned to the left (‘E’) key and ‘Not Me’ with the right (‘I’) key. On the second block of 20 practice trials participants had to assign positively valenced stimuli to the ‘Worth’ category using the left key and negative stimuli to the ‘Worthless’ category using the right key. Blocks 3 (20 trials) and 4 (40 trials) involved a combined assignment of target and attribute stimuli to their respective categories. Specifically, participants categorized ‘Me’ and positive words using the left key and ‘Not Me’ and negative words using the right key. The fifth block of 20 trials reversed the key assignments for self- and other-related items, with ‘Me’ now assigned to the right key and ‘Not Me’ with the left key. Finally, the sixth block (20 trials) and seventh block (40 trials) required participants to categorize ‘Me’ and negative words with the right key and ‘Not Me’ and positive words with the left key. The order of the critical test blocks was counterbalanced across participants.

#### IRAP

The IRAP is a computerized latency-based measure which requires participants to respond quickly and accurately to stimuli in ways that are deemed consistent or inconsistent with their prior learning history. Specifically, half of the IRAP trials require participants to respond in ways that are consistent with their (assumed) history of learning, while the other half require participants to respond in ways that are inconsistent with that same history. For instance, participants might be asked to respond “True” to the statement “*I want to be Good*” on half of the trials but to respond “False” on the other half. The difference in time taken to respond on consistent relative to inconsistent trials - defined as the IRAP effect - is assumed to provide an index of the strength or probability of the targeted relations. Reliability estimates differ substantially between studies, ranging from values as low as.23 to values as high as.81 (for more on the measure and its psychometric properties see [37], [38]).

**Figure 1:**
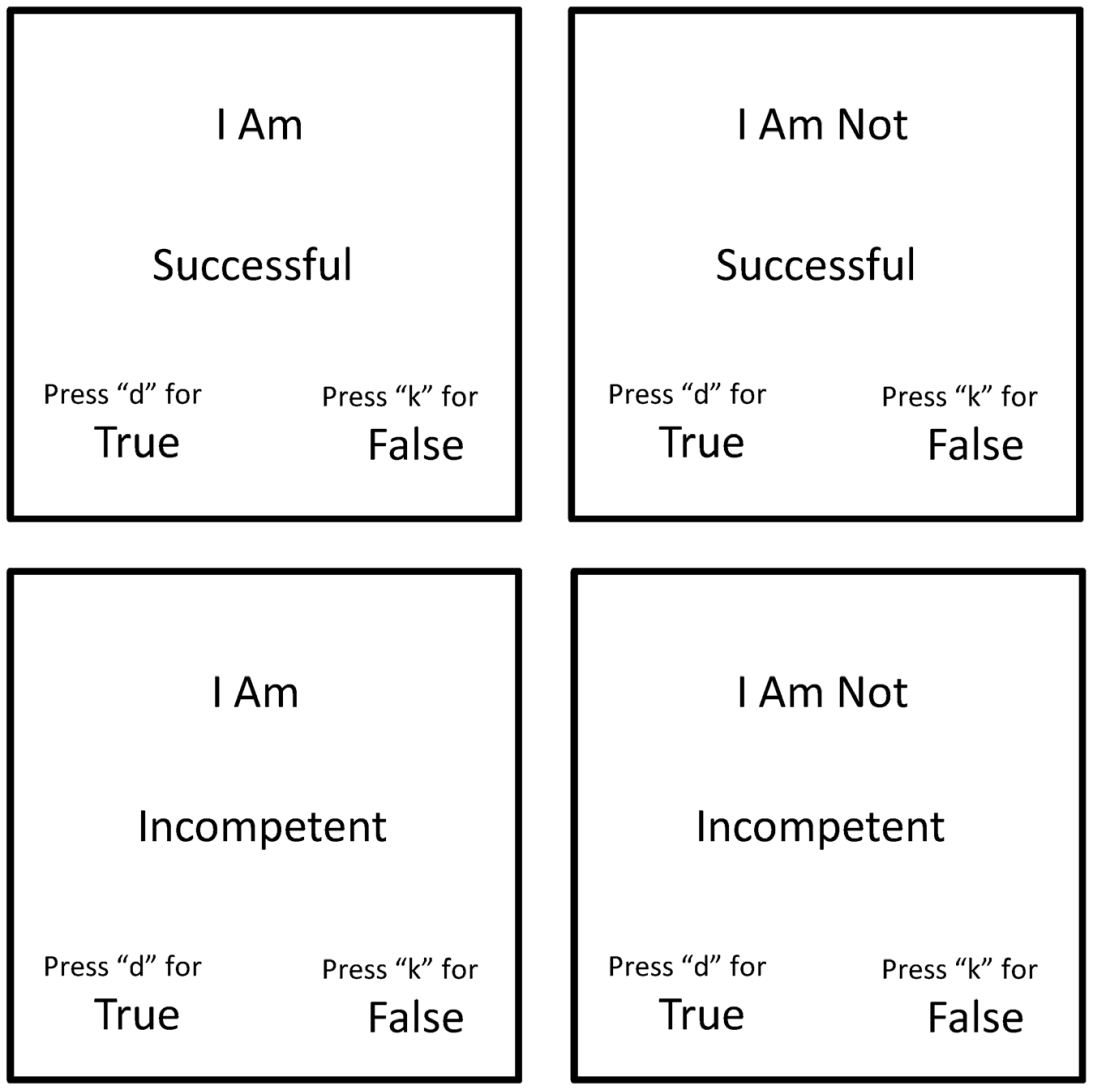
Examples of the four trial-types used in the actual self-esteem IRAP. On each trial, a label stimulus (e.g., ‘I am’ or ‘I am not’), a target stimulus (e.g., ‘Successful’ or ‘Incompetent’) and two relational response options (True and False) were shown on the screen. Note: the ideal and actual self IRAPs were identical in all regards except for their respective label stimuli (‘I want to be’ and ‘I don’t want to be’ versus ‘I am’ and ‘I am not’ respectively).

In the current study, each IRAP involved a minimum of two and a maximum of six practice blocks followed by a fixed set of six test blocks. Each block consisted of 24 trials that presented one of two self-related label stimuli (e.g., ‘*I Am*’ or ‘*I Am Not*’) in the presence of one of two types of target stimuli (positive or negative words drawn from the same set as the IAT) and required participants to emit one of two relational responses (‘True’ or ‘False’). In this way, the IRAP was comprised of four different types of trials (or “trial-types”: *Self-Positive*; *Self-Not Positive*, *Self-Negative* and *Self-Not Negative*; see Figure 1). Trials were presented in a quasi-random order so that each of the four trial-types appeared six times within each block in a random order.

Prior to the IRAP participants were informed that they would complete a word categorization procedure that required them to follow a general rule for responding. Specifically, on one set of blocks they were presented with the message “*Please respond AS IF I am positive and I am not negative*” (self-positive block), while on the alternative set of blocks they were presented with the message “*Please respond AS IF I am negative and I am not positive*” (self-negative block). Stated more precisely, a correct response during self-positive blocks required participants to select ‘True’ when ‘I Am’ appeared with a positive target stimulus (e.g., ‘*Intelligent*’) or when ‘I Am Not’ appeared with a negative target (e.g., ‘*Stupid*’). At the same time, participants were also required to choose ‘False’ when ‘I Am’ appeared with a negative word or when ‘I Am Not’ appeared with a positive target stimulus. The opposite pattern of responding was required during self-negative blocks. The general rule for responding was alternated across each IRAP block to form three successive pairs of test blocks.

The IRAP commenced with a pair of practice blocks. Participants progressed from the practice to the test blocks when they met accuracy (at least 80% accuracy) and latency criteria (median latency of less than 2000 ms) on a successive pair of practice blocks. Failure to meet these criteria resulted in re-exposure to another pair of practice blocks until participants either achieved the mastery criteria or a maximum of three pairs of practice block were completed. Failure to satisfy task requirements following three pairs of practice blocks resulted in participants being thanked, debriefed and dismissed (in the current study one participant failed to complete both IRAPs, another three failed the actual self IRAP while six more did not satisfy those same criteria during the ideal self IRAP). When the above criteria were met, a fixed set of three pairs of test blocks were then administered. Finally, it is worth noting that the actual and ideal self IRAPs differed only with respect to their self-related label stimuli. That is, while the actual self IRAP required participants to respond to valenced target stimuli using the terms ‘*I Am*’ or ‘*I Am Not*’ the ideal self IRAP required participants respond to the same stimuli in terms of ‘*I Want To Be*’ or ‘*I Don’t Want To Be*’.

### Procedure

Upon arriving at the laboratory participants were welcomed by the researcher, asked to read and sign statements of consent and seated in front of a computer from which they received all instructions. They were informed that they would complete a number of questionnaires as well as computer based tasks - and given the sensitive nature of the study - that they would be randomly assigned an identification number in order to preserve their confidentiality and anonymity. Thereafter, participants completed the various self-report measures, an IAT and two IRAPs. The order of questionnaires and implicit measures as well as the order of the two IRAPs were counterbalanced across participants. The IAT was always administered prior to the two IRAPs. Overall, the experiment lasted about 60 minutes.

## Results

### Data Preparation

Counterbalancing the order of the two IRAPs as well as evaluative measures (questionnaires and implicit measures) did not produce any main or interaction effects. Consequently, data were collapsed across both factors.

### Implicit Measures

#### IAT

Following the recommendations of Greenwald and colleagues [39], response latency data from the IAT was prepared using the D1 scoring algorithm. This transformation resulted in one IAT score for each participant, reflecting the difference in mean response latency between consistent and inconsistent blocks divided by the overall variation in those latencies. Scores were calculated so that positive values reflected a relatively higher positive self-esteem bias whereas negative values indicated the opposite. When IAT scores from the dysphoric and non-dysphoric groups were submitted to an independent samples t-test no significant difference emerged, *t*(62) = .81, *p* = .42. Consistent with our predictions, dysphoric (*M* = .59, *SD* = .47) and non-dysphoric groups (*M* = .68, *SD* = .35) both demonstrated similar and robust levels of positive implicit self-esteem.

#### IRAP

**Figure 2:**
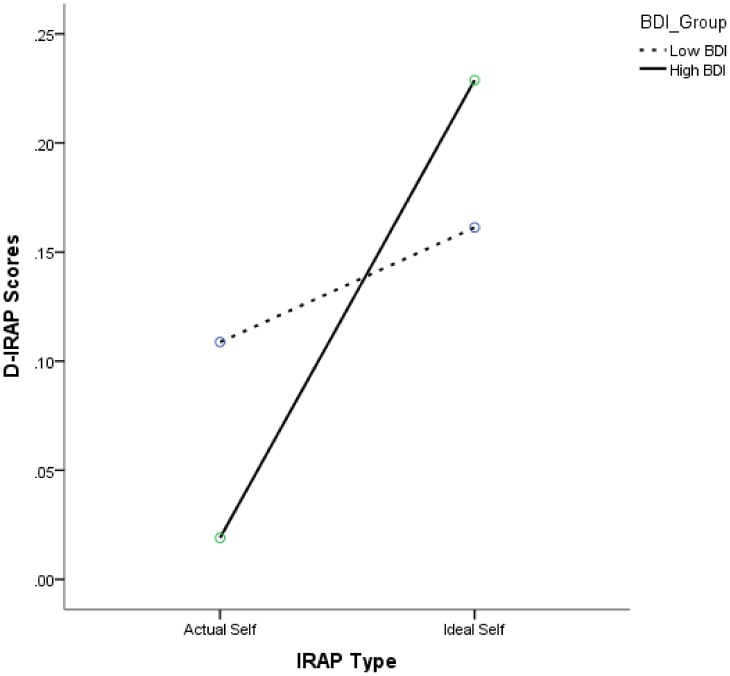
Mean D-IRAP scores as a function of IRAP-Type (actual vs. ideal) and BDI group (high vs. low). A positive value indicates a pro self-esteem bias and a negative score indicates the opposite.

Response latency data were transformed into *D*-IRAP scores using an adaptation of Greenwald et al. 's [39] *D* algorithm (for details of this data transformation see [26]). For each IRAP, we calculated a single overall *D*-IRAP score - one for the actual self IRAP and a second for the ideal self IRAP. These values were calculated so that higher scores reflected higher levels of (actual or ideal) self-esteem. When submitted to a 2 (*BDI Group*) x 2 (*IRAP-Type;* Actual vs. Ideal) mixed-models ANOVA, a main effect for IRAP-Type, *F*(1, 52) = 14.72, *p<*.001, *η^2^_partial_* = .22, as well as a two-way interaction between IRAP-Type and BDI Group was obtained, *F*(1, 52) = 5.29, *p = *.03, *η^2^_partial_* = .09. This crucial interaction effect reveals a stronger discrepancy between actual and ideal self-esteem IRAP scores in dysphoric participants (*M* = .21, *SD* = .29) than in non-dysphoric participants (*M* = .05, *SD* = .19),

To explore this interaction, we compared BDI groups for each IRAP separately as well as both IRAPs for each group separately. The first set of analyses did not reveal differences between the dysphoric and non-dysphoric groups in terms of their respective IRAP performances (all *ps*>.2). The second set of analysis did not reveal a difference between scores on the actual (*M* = .11, *SD* = .27) and ideal (*M* = .16, *SD* = .24) IRAPs for non-dysphorics (*p*>.2) but did reveal more positive scores on the ideal self (*M* = .23, *SD* = .24) relative to the actual self IRAP (*M* = .02, *SD* = .22) for dysphoric participants, *t*(25) = 3.6, *p* = .001, *d = *.93 (see Figure 2). To assess the internal consistency of the IRAP, two split-half reliability scores were calculated, one for the actual self IRAP and one for the ideal self IRAP. In each case, two scores were calculated, one for odd trials and the second for even trials, and these were obtained in the same way as for the overall *D*-IRAP score, except that the *D*-algorithm was applied separately to all odd trials and even trials. The split-half correlations between odd and even scores, applying Spearman-Brown corrections, for the Actual-Self IRAP was (*r* = .53) and Ideal-Self IRAP was (*r* = .45). These split-half reliabilities were based on all participants who completed both IRAPs. The IAT's internal consistency (*r* = .96) was based on a Spearman-Brown corrected split-half correlation, the split-halves being derived from alternating pairs of trials in both critical blocks

### Explicit Measures

Consistent with our predictions, we found that dysphoric participants (*M* = 13.0, *SD* = 3.0) showed significantly lower self-esteem scores on the Rosenberg scale relative to their non-dysphoric counterparts (*M* = 20.6, *SD* = 3.5), *t*(62) = 9.11, *p<*.001, *d* = 2.29. When actual and ideal-self evaluations were submitted to a 2 (*Self*: Actual vs. Ideal) x 2 (*BDI Group*) mixed models ANOVA, a main effect for BDI Group, *F*(1, 62) = 48.13, *p<*.001, *η^2^_partial_* = .44, and a two-way interaction between Self and BDI Group was obtained, *F*(1, 62) = 50.99, *p<*.001, *η^2^_partial_* = .45. This reveals that dysphoric participants showed significantly higher self-discrepancy scores (*M* = 19.83, *SD* = 6.60) than their non-dysphoric counterparts (*M* = 9.91, *SD* = 4.45). To explore this interaction, we compared BDI groups for each self-evaluation separately as well as both self-evaluations for each group separately. The first set of analyses revealed that non-dysphoric participants (*M* = 35.14, *SD* = 4.72) reported significantly higher actual self-evaluations than their dysphoric counterparts (*M* = 24.24, *SD* = 6.03), *t*(62) = 8.11, *p*<.001. Dysphoric (*M* = 44.07, *SD* = .34) and non-dysphoric individuals (*M* = 45.06, *SD* = 2.89) showed similar and high levels of ideal-self evaluations (*p* = .22). The second set of analysis revealed a significant difference between actual and ideal self-evaluations for both dysphoric, *t*(28) = 16.18, *p* = .001, and non-dysphoric participants, *t*(35) = 13.17, *p* = .001.

### Correlations

#### Implicit-explicit correlations

In the non-dysphoric group, the IAT and ideal self-evaluations (SR Ideal) correlated positively, *r* = 0.43, n = 35, *p* = .009, while a marginally significant positive correlation appeared between the IAT and actual self-evaluations (SR Actual), *r* = 0.30, n = 35, *p* = .077. However, no significant correlations emerged between the actual and ideal IRAPs and any of the explicit measures. With respect to the dysphoric group, no significant correlations emerged between the IAT and the various explicit measures. However, the actual (but not the ideal self IRAP) correlated positively with self-esteem (RSES), *r* = 0.42, n = 28, *p* = .027, and actual self-evaluations (SR Actual), *r* = 0.53, n = 28, *p* = .004.

#### Implicit-Implicit correlations

A series of correlations within dysphoric and non-dysphoric participants were used to determine whether IAT and IRAP effects were related but none of the tests proved significant (see Tables 1 and 2): IAT with actual self IRAP (all *ps>*.3); IAT with ideal self IRAP, (all *ps>*.6). A significant correlation did emerge between the actual and ideal self IRAPs for the non-dysphoric, *r* = .70, n = 28, *p<*.001, but not the dysphoric group (*p* = .51). Although participants were pre-selected because they had high or low scores on the BDI during a screening study, a number of individuals nevertheless revealed BDI scores around the cut-off point during the actual test session. When a more stringent cut-off value was employed to create the non-dysphoric (scores from 0–9) and dysphoric groups (scores from 16–64) an almost identical set of findings emerged.

**Table 1:** Correlation matrix of explicit and implicit self-esteem scores for the low BDI group.

	IAT	Actual IRAP	Ideal IRAP	RSES	SR Actual	SR Ideal
IAT						
Actual IRAP	.20					
Ideal IRAP	.10	.70**				
RSES	−.03	−.23	.05			
SR Actual	.30	.04	.05	.55**		
SR Ideal	.43*	.17	.20	.01	.40*	

Note: RSES  =  Rosenberg Self-esteem Scale; SE Actual  =  Self-reported actual self-esteem; SR Ideal  =  Self-reported ideal self-esteem. * = *p*<.05 ** = *p*<.001.

**Table 2:** Correlation matrix of explicit and implicit self-esteem scores for the high BDI group.

	IAT	Actual IRAP	Ideal IRAP	RSES	SR Actual	SR Ideal
IAT						
Actual IRAP	.02					
Ideal IRAP	.03	.13				
RSES	.28	.42*	−.01			
SR Actual	.20	.53*	−.06	.71**		
SR Ideal	.09	−.31	.06	.00	.11	

Note: RSES  =  Rosenberg Self-esteem Scale; SE Actual  =  Self-reported actual self-esteem; SR Ideal  =  Self-reported ideal self-esteem. * = *p*<.05 ** = *p*<.001.

#### Explicits

In the non-dysphoric group, we found a significant positive correlation between self-esteem (RSES) and actual (SR Actual), *r* = 0.56, n = 35, *p* = .001. Finally, actual (SR Actual) and ideal (SR Ideal) self-esteem correlated positively, *r* = 0.40, n = 35, *p* = .019. With respect to the dysphoric group, self-esteem (RSES) and actual self-evaluations (SR Actual) correlated positively, *r* = 0.71, n = 29, *p<*.001 (see Tables 1 and 2).

## Discussion

Accumulating evidence suggests that although depressed and non-depressed people differ with respect to their explicit self-esteem they demonstrate surprisingly similar levels of (positive) implicit self-esteem. In an attempt to explain these surprising findings, it has been argued that the IAT and other implicit measures capture *actual* self-esteem in non-depressed participants but *ideal* self-esteem in depressed participants [11], [22]. In the current study we put this assumption to the test. In particular, we examined whether implicit measures designed to capture associations between the self and valenced stimuli (IAT) actually reflect the operation of qualitatively distinct sets of self-related propositions (IRAP). Whereas De Raedt and colleagues [11] only used an IAT and Remue et al. [22] only used IRAPs, we asked participants to complete both a self-esteem IAT and two separate IRAPs, one targeting actual (‘*I am*’) and another targeting ideal self-evaluations (‘*I want to be*’). Based on previous work, we expected to observe three outcomes. First, dysphoric and non-dysphoric participants should produce similar (positive) scores on the self-esteem IAT. Second, those same participants should diverge in their respective IRAP performances, with dysphorics showing stronger ideal relative to the actual self-esteem and non-dysphorics stronger actual relative to ideal self-esteem. Third, performance on the actual-self IRAP (in the non-dysphoric group) and performance on the ideal-self IRAP (in the dysphoric group) should differentially correlate with the IAT.

Consistent with our first prediction, we found that dysphoric and non-dysphoric participants were relatively quicker to categorize self-related words with positive compared to negative stimuli on the IAT. This finding is also consistent with work elsewhere in the literature on the near universal positivity towards the self [14] that seems to emerge regardless of current or former depressive symptomatology [15], [16]. At the same time, our results extend beyond this early work. As indicated by the significant interaction between IRAP type and group, dysphoric participants showed a greater discrepancy between their (implicit) actual and ideal self-esteem than their non-dysphoric counterparts. This result replicates the crucial finding of Remue and colleagues [22]. However, several caveats should be noted. First, although the interaction between IRAP type and group was significant, several of the simple main effects involved in this interaction did not reach conventional levels of significance. Whereas dysphorics did show higher scores on the ideal self-esteem IRAP than on the actual self-esteem IRAP, non-dysphorics did not score differently on the two IRAPs. Hence, we did not replicate the finding of Remue et al. that non-dysphorics have a higher score on the actual self-esteem IRAP than on the ideal self-esteem IRAP. Unlike Remue et al., we also did not observe significant differences between groups in their performance on each of the IRAPs. Finally, and contrary to our third prediction, we did not observe a contrasting pattern of correlations between the IAT and IRAP as a function of depressive symptomatology.

Although our main goal was to investigate differences between different types of implicit self-esteem, we also included a number of questionnaires in order to investigate explicit self-esteem, and its relationship with implicit self-esteem. We found that dysphoric participants produced significantly lower scores on the Rosenberg scale relative to non-dysphoric participants. However, when actual and ideal-self evaluations were compared, a more complex picture emerged. Both groups displayed higher levels of ideal relative to actual-self evaluations, with the dysphoric group producing significantly lower actual-self scores than their non-dysphoric peers. Following the discrepancy theory of Higgins [18] which states that the discrepancy between the actual and ideal self is a cognitive risk factor for depression, and consistent with previous work in this area (e.g., [40]), individuals suffering from higher levels of self-reported depressive symptomatology displayed greater discrepancies between their ideal and actual self-evaluations than those who did not report such symptoms. Note that discrepancy theory is supported not only by the effects that we observed on the explicit measures but also by the differences between groups in actual-ideal self-esteem discrepancy on the implicit measures.

We also found that implicit and explicit self-esteem correlated with one another in different ways as a function of depressive symptomatology. For instance, actual and ideal-self evaluations in the non-dysphoric condition tended to correlate regardless of the measure used. That is, explicit measures of ‘actual’ self-esteem correlated with explicit ‘ideal’ self-esteem while both explicit measures correlated with performance on the IAT in the non-dysphoric group. However, no correlations emerged between actual and ideal self-evaluations on either the explicit or implicit measures for participants in the dysphoric group.

Based on the above, an important next step is to develop a more sophisticated understanding of how self-related cognitions impact implicit and explicit self-esteem. In conducting this work several points are worth noting. First, the research presented here (as well as in Remue et al. [22]) utilized a normative sample of students that varied in their respective levels of self-reported depressive symptomatology. It remains to be seen whether a sample of clinically depressed, remitted or recovered participants would also show evidence of elevated ideal and diminished actual self-evaluations. Second, it may be that other implicit propositions such as those related to people's personal expectations (e.g., ‘*I should be*’ or ‘*I need to be*’), how they compare themselves to others (e.g., ‘*I am good but others are better*’) or perceived failures (e.g., ‘*I’m not good enough*’) are even more important for predicting behavior. With this in mind, research could examine whether IRAPs targeting other types of propositional knowledge provide even better diagnostic and predictive information about clinical and non-clinical populations. Third, while the current study assessed propositions related to actual and ideal self-esteem separately via two IRAPs, it may be that juxtaposing one set of propositions (e.g., ‘*I am good*’) with another (e.g., ‘*I need to be better*’) within a single IRAP would enable us to determine how the assessment context influences the activation of different propositions and their respective influence on one another. It may be that activating two sets of propositions within rather than across measurement contexts could magnify discrepancies between actual and ideal self-evaluations.

To the best of our knowledge, we are the first to test the idea that a single IAT might actually reflect different implicit beliefs in different people. More specifically, the fact that dysphoric and non-dysphoric individuals reveal similarly high scores on IAT it might be due to the fact that the IAT reflects (high) ideal self-esteem in dysphorics and (high) actual-self esteem in non-dysphorics. Based on this idea, we predicted that IAT scores should correlate primarily with ideal self-esteem IRAP scores in dysphorics but with actual self-esteem IRAP scores in non-dysphorics. Our data do not, however, reveal such pattern of correlations. Although these null findings might indicate that the IAT does not capture different beliefs in different groups, it is also possible other factors came into play. First, IRAPs scores were somewhat unreliable which reduces changes of finding meaningful correlations. Second, counterbalancing of the order of the three tasks and administrating those three task within a single session could have increased error variance.

Finally, in replicating the work of Remue and colleagues [22], we implemented a number of methodological refinements that sought to strengthen the arguments forwarded in that earlier paper (e.g., we used a traditional six-block variant of the IRAP, more stringent mastery criteria and stimulus selection). In their paper, a number of dysphoric and non-dysphoric participants (22%) failed to complete an IRAP and they may have done so for entirely different reasons, with the former failing due to a lack of motivation and the latter due to an inability to respond quickly and accurately to certain propositions (or even vice-versa). The modifications implemented in the current study appear to be successful insofar attrition rates (14%) were lower than those reported by Remue et al. and other studies elsewhere in the IRAP literature (see [29]). In addition, the split-half reliability estimates obtained in the current study proved to be relatively higher then to those seen in Remue et al. and elsewhere in the literature.

Although we did observe a significant interaction between group and IRAP type, other effects failed to reach significance (e.g., lack of group difference on the IAT and the two IRAPs). In part, these null effects could be due to a lack of power because of the relatively small sample. We therefore recommend that replications of our findings - especially those comparing clinical and healthy populations - incorporate power analyses to ensure that an adequate sample size is employed so that statistically reliable inferences can be drawn. The lack of power could also explain why we failed to replicate the observation of Remue et al. that non-dysphorics score higher on the actual self-esteem IRAP than on the ideal self-esteem IRAP, as well as the observation that both groups differed in their performance on each of the IRAPs. Nevertheless, future work could explore whether differences in the number of IRAP blocks, stimuli employed, mastery criteria used or other procedural properties contribute to the inconsistencies observed between the results of our study and the results of Remue et al. For instance, we always exposed participants to an IAT before the two IRAPs, which may have influenced the expression of self-related evaluations on the IRAP. Future work could counterbalance these measures to assess potential carry-over effects between measures.

## Conclusion

To summarize, our results indicate that dysphoric and non-dysphoric individuals experience implicit positivity towards the self. Most importantly, dysphoric participants revealed a stronger discrepancy between actual and ideal self-esteem as indexed by IRAPs compared to non-dysphoric participants. This finding not only supports the theoretical position that the discrepancy between actual and ideal self-esteem is related to dysphoria but also demonstrates the added value of using implicit measures such as the IRAP that can capture different implicit beliefs.
